# Study on radiation damage of tungsten-based targets induced by deuterium ions

**DOI:** 10.1371/journal.pone.0360154

**Published:** 2026-10-14

**Authors:** Yanli Zhang, Fei Zheng, Jiangping Feng, Zhongsheng Cao, Qingyun Meng

**Affiliations:** 1 College of Mechanical, Electrical and Aerospace Engineering, Xichang University, Sichuan, China; 2 Department of Nuclear Energy, The Engineering and Technical College of Chengdu University of Technology, Sichuan, China; 3 School of Mathematics and Physics, Beijing University of Chemical Technology, Beijing, China; Pacific Northwest National Laboratory, UNITED STATES OF AMERICA

## Abstract

The effects of homogeneously doped phases on the primary radiation damage of W-based targets were investigated by the Monte Carlo method in this paper. The results show that introducing homogeneously doped phases into W effectively reduces the energy localization of deuterium ions within the targets. Meanwhile, Under 100 keV deuterium ion irradiation, the ballistic distribution, the number of displacement collisions, and the vacancies of deuterium ions in homogeneously doped W targets are all lower than those in pure W. And longitudinal analysis along the depth of all homogeneously doped specimens confirms that homogeneously TiC-doped W offers the strongest suppression of primary radiation damage in the near-surface region. Under 200 keV deuterium ion irradiation, the governing effects of various dopant species and dopant contents on primary radiation damage are summarized as follows: increasing dopant content leads to an approximately linear reduction in vacancies, with Ti dopant showing the strongest vacancy inhibition effect, while the ion range rises linearly and W–TiC possesses the most significant range variation. Large sampling fluctuations of sputtering yield make it an unreliable indicator for evaluating doping performance. Overall, dopants effectively mitigate primary irradiation damage in tungsten. These volume-averaged primary irradiation damage results derived from SRIM simulation are expected to offer reference insights into improving the irradiation tolerance of W by optimizing their compositional design, while microstructural interface effects need further multi-scale modeling for quantitative analysis.

## 1. Introduction

Controlled thermonuclear fusion energy is regarded as the ultimate solution to future energy challenges for humankind [1], and it is still in the research stage. Many factors affect its commercialization, among which materials issues are one of the core constraints, especially plasma-facing first-wall materials [2,3]. Such materials work under extreme conditions, yet no first-wall material can fully meet the operational requirements of fusion reactors at present.

Tungsten (W), owing to its high melting point, high thermal conductivity, low sputtering yield, low hydrogen retention, and low neutron activation, is considered one of the leading candidates for first-wall materials in fusion reactors [4–8]. It has been widely applied in the International Thermonuclear Experimental Reactor (ITER) [9], the Joint European Torus (JET) [10], the ASDEX Upgrade (ASDEX-U) tokamak in Germany [11], the WEST tokamak in France [12], and the Experimental Advanced Superconducting Tokamak (EAST) in China [13], among other experimental devices [14]. However, W still faces critical challenges during service, including radiation damage, low-temperature brittleness, and recrystallization embrittlement [15–18]. To improve tungsten’s suitability for fusion reactors, extensive studies have been carried out worldwide on microstructural optimization and compositional design. A typical technical route is dispersion strengthening: adding alloying elements such as titanium (Ti) [19,20], rhenium (Re) [21,22], and chromium–yttrium (Cr–Y) [23–26], or introducing high-melting-point carbides (TiC [27,28], ZrC [29], HfC [30], etc.) and oxides (La_2_O_3_ [31–33], Y_2_O_3_ [34,35], etc.) as particles to obtain a uniform dispersion within the tungsten matrix. These particles can effectively adsorb impurities in tungsten, thereby reducing impurity segregation and providing dual functions of grain-boundary purification and strengthening. In addition, the particles pin grain boundaries and inhibit tungsten grain growth, thus improving strength and toughness. Studies have shown that such ultrafine-grained dispersion-strengthened materials not only exhibit significantly enhanced strength and toughness, but their dopant/matrix interfaces can also annihilate irradiation-induced point defects, thereby improving irradiation resistance and showing excellent irradiation tolerance [14,36,37]. Therefore, developing high-strength and high-toughness dispersion-strengthened tungsten has become a key direction for future fusion-reactor first-wall materials.

Radiation damage in materials is a complex kinetic process that involves three stages: primary radiation damage, micro- and meso-scale defect evolution, and macroscopic damage. It spans time scales from femtoseconds to years (20 orders of magnitude) and spatial scales from nanometers to meters (9 orders of magnitude). Primary radiation damage occurs at the picosecond (ps) time scale and nanometer (nm) spatial scale, generating atomic-level defects such as self-interstitial atoms, vacancies, and clusters. Accurately describing primary radiation damage is not only the key to understanding how irradiation behavior varies with parameters, but also the basis for predicting macroscopic damage and in-service performance. Because primary radiation damage is extremely short-lived and occurs on a very small spatial scale (only hundreds of nm^3^), it is difficult to track directly by experiments; therefore, revealing its microscopic mechanisms requires computational simulations. Currently, numerous studies have investigated defect formation, growth, and evolution using molecular dynamics and density functional theory [38–44], but research specifically targeting primary radiation damage remains relatively limited. SRIM-2013 is a classic irradiation-damage simulation software widely used in irradiation-related studies [45–51]. In this work, the computational methodology of SRIM-2013 is applied to study primary radiation damage induced by deuterium ions in various homogeneously doped tungsten-based targets, aiming to provide theoretical references for irradiation performance, as well as guidance for formulation optimization and irradiation lifetime prediction.

It is necessary to clarify the inherent simplifying assumptions of the Binary Collision Approximation (BCA) model implemented in SRIM 2013. Within this simulation framework, all multi-phase targets (W-Ti, W-La_2_O_3_, W-TiC, W-ZrC) are treated as statistically homogeneous continuous media calculated with homogenized average bulk density. The model fails to resolve microstructural heterogeneities such as grain boundaries, discrete dopant particles, matrix-particle phase interfaces, and local compositional segregation. In addition, SRIM only computes instantaneous primary ballistic radiation damage generated at the moment of ion impact, without accounting for long-term evolutionary processes including defect recombination and thermal relaxation. Accordingly, all calculated vacancy counts in this work represent statistical primary damage outputs under the homogenized medium assumption, which cannot be equated to the actual stable vacancy governing long-timescale microstructural degradation. Meanwhile, the present SRIM simulation cannot capture the local evolution of irradiation defects triggered by microscale interfaces.The volume-averaged primary irradiation damage results obtained from SRIM BCA simulations can provide referential insights for enhancing the irradiation resistance of tungsten-based materials via compositional optimization. Quantitative analysis of microstructural interface effects and post-cascade long-term defect evolution, however, requires further multi-scale modeling.

## 2. Research methodology and parameter settings

The present study is mainly based on the Monte Carlo method and the binary collision approximation (BCA). By randomly sampling binary collisions between ions and atomic nuclei or between ions and extranuclear electrons, ion trajectories and macroscopic quantities—such as energy loss, vacancy production, sputtering yield, and range—can be obtained to evaluate ion-induced radiation damage in materials. The methodology involves two core issues: binary collisions between ions and target nuclei, and binary interactions between ions and extranuclear electrons. For ion–nucleus collisions, the Ziegler–Biersack–Littmark (ZBL) screened Coulomb interaction potential [52] is adopted. The potential is expressed as:


$$\begin{array}{c} \text{v}(\text{r})=\frac{{\text{Z}}_{\text{1}}{\text{Z}}_{\text{2}}{\text{e}}^{\text{2}}}{\text{4}\pi \varepsilon_{\text{0}}\text{r}}(\text{0}.\text{1818}{\text{e}}^{-\text{3}.\text{2}\frac{\text{r}}{\text{a}}}+\text{0}.\text{5099}{\text{e}}^{-\text{0}.\text{9423}\frac{\text{r}}{\text{a}}}+\text{0}.\text{2802}{\text{e}}^{-\text{0}.\text{4028}\frac{\text{r}}{\text{a}}}+\text{0}.\text{02817}{\text{e}}^{-\text{0}.\text{2016}\frac{\text{r}}{\text{a}}}) \end{array}$$ (1)

where *a* is the ZBL screening length, representing the effective interaction range of the potential. Based on the ZBL screened Coulomb potential and collision parameters, classical scattering theory is used to calculate the post-collision scattering angle of the ion, the energy loss, and the energy transferred to target atoms.

For ion–electron interactions, a semi-empirical model is employed. Using experimentally measured proton stopping-power data as a reference, this model incorporates an effective charge model and a charge-oscillation correction factor. The electronic stopping power is obtained by solving Eq. (2):


$$\begin{array}{c} S_{e}(E)={\left(\frac{Z_{\text{1}}^{*}}{Z_{\text{1}}}\right)}^{\text{2}}.S_{e.proton}(E).L \end{array}$$ (2)

where *Z*_1_* is the effective charge and *L* is the oscillation correction factor. The ion position, energy, and direction are repeatedly updated using Eqs. (1) and (2) until the ion energy falls below a threshold value or the ion exits the target. By statistically averaging over a large number of ions, physical quantities such as range distribution, energy deposition, vacancy production, and sputtering yield are obtained.

To investigate the effects of different dopants on irradiation performance of tungsten-based materials, five targets were selected: pure W, W–Ti, W–La_2_O_3_, W–TiC, and W–ZrC. The incident particles were deuterium ions with energies ranging from 10 eV to 10 MeV. Simulations were performed using SRIM 2013. Two key parameters—the displacement threshold energy and the surface binding energy—must be specified, because they directly affect the accuracy of vacancy production and sputtering calculations. Parameter values are listed in Table 1. The surface binding energies were set to the software default values. For polycrystalline tungsten, the displacement threshold energy adopts the full-orientation full-temperature average value of 61.47 eV from MD data in Ref. [46]; The full-range averaged threshold displacement energies adopted for other phases are listed as follows: 78.30 eV for Ti (Ref. [53]); 90.70 eV for La and 44.20 eV for O in La_2_O_3_ (Ref. [54]); 52.25 eV for Ti and 32.13 eV for C in TiC (Ref. [55]); 47.75 eV for Zr and 34.08 eV for C in ZrC (Ref. [56]). The number of incident ions was set to the software default maximum value of 99,999. To accurately capture the complete collision process inside composite tungsten materials, the calculation mode was set to “Detailed Calculation with Full Damage Cascades”.

**Table 1:** Displacement threshold energies and surface binding energies of components in tungsten-based targets.

Components	Displacement threshold energy/eV	Surface binding energy/eV
W	61.47^b^	8.68^a^
Ti	78.30^c^	4.89^a^
La_2_O_3_	La: 90.70^d^ O: 44.20^d^	La: 4.42^a^ O: 2.00^a^
TiC	Ti: 52.25^e^ C: 32.13^e^	Ti: 4.89^a^ C: 7.41^a^
ZrC	Zr: 47.75^f^ C: 34.08^f^	Zr: 6.33^a^ C: 7.41^a^

^a^Surface binding energies were assigned the default values provided by SRIM software.

^b^Value is averaged from the corresponding data in Ref. [45].

^c^Value is derived from Ref. [53].

^d^Values is derived from Ref. [54].

^e^Values is derived from Ref. [55].

^f^Values is derived from Ref. [56].

All dopant concentrations in this work are expressed in weight percent (wt.%). For the SRIM calculations, each homogeneously doped target (W-Ti, W-La_2_O_3_, W-TiC, and W-ZrC) was defined as a compound target in SRIM. The nominal composition was converted from mass fractions to atomic fractions using the molar masses of the constituent elements, and the atomic fractions were then used as the input parameters of the SRIM compound target. The density of each target was automatically calculated by SRIM from its built-in density database via the rule of mixtures, without manual density adjustment. To improve the reproducibility of our simulations, The corresponding conversion table of mass fractions to atomic fractions used for SRIM input parameters is provided in Supporting Information (S9 File).

In this study, the full damage cascades mode was adopted for irradiation damage calculations. This mode can fully track the transport and collision behaviors of all generations of recoil atoms to accurately reproduce the evolution of collision cascades, and it enables quantitative calculation of sputtering yield, which meets the research requirements of this work. Comparative simulations under identical simulation conditions reveal that this method yields more adequate vacancy quantities. Furthermore, the full damage cascades mode exhibits good sensitivity to variations in target composition; the number of vacancies changes regularly with dopant fraction, which can effectively support the analysis of the correlation mechanism between vacancy evolution and dopant content.

It should be noted that SRIM provides two damage-calculation modes: the “Detailed Calculation with Full Damage Cascades” (FDC) mode and the “Norgett-Robinson-Torrens (NRT) -standard Quick Damage mode”. The predicted vacancy numbers and their relative trends are dependent on the calculation mode chosen. All calculation results and rankings reported in this work are obtained from FDC simulation, since FDC can reproduce full-order recoil collision cascades, calculate sputtering yield and capture composition-dependent vacancy variations required for our analysis, while NRT-standard Quick Damage mode relies only on analytical estimation, excludes sputtering-yield output and exhibits low sensitivity to target-composition changes. For completeness, a comparison between the vacancy results obtained with the FDC and NRT modes is provided in the Supporting Information (S10 File).

## 3. Results and discussion

### 3.1. Stopping power

Stopping power directly determines the energy-loss rate and the total energy loss of deuterium ions in targets: a higher stopping power results in faster energy loss and a larger total loss. To identify the dominant energy-loss channel of deuterium ions in W-based targets, Fig 1 shows the electronic stopping power and nuclear stopping power as functions of incident energy for the five targets. As shown in Fig 1, for all five targets the electronic stopping power is larger than the nuclear stopping power, indicating that deuterium-ion energy loss in these targets is dominated by ionization (electronic) energy loss. When the ion energy E < 250 keV, the electronic stopping power increases rapidly with energy and reaches a maximum at E ≈ 250 keV; it then decreases approximately exponentially with further increasing energy and gradually approaches a stable value at sufficiently high energies. The nuclear stopping power varies only weakly with ion energy and remains approximately constant. The stopping-power trends are similar for all targets; among them, the electronic stopping power of TiC homogeneously doped W is slightly higher than that of the other targets, this conclusion is consistent with the finding in Ref. [57] that TiC increases the proportion of electronic energy loss and weakens the intensity of nuclear collision cascades.

**Fig 1:**
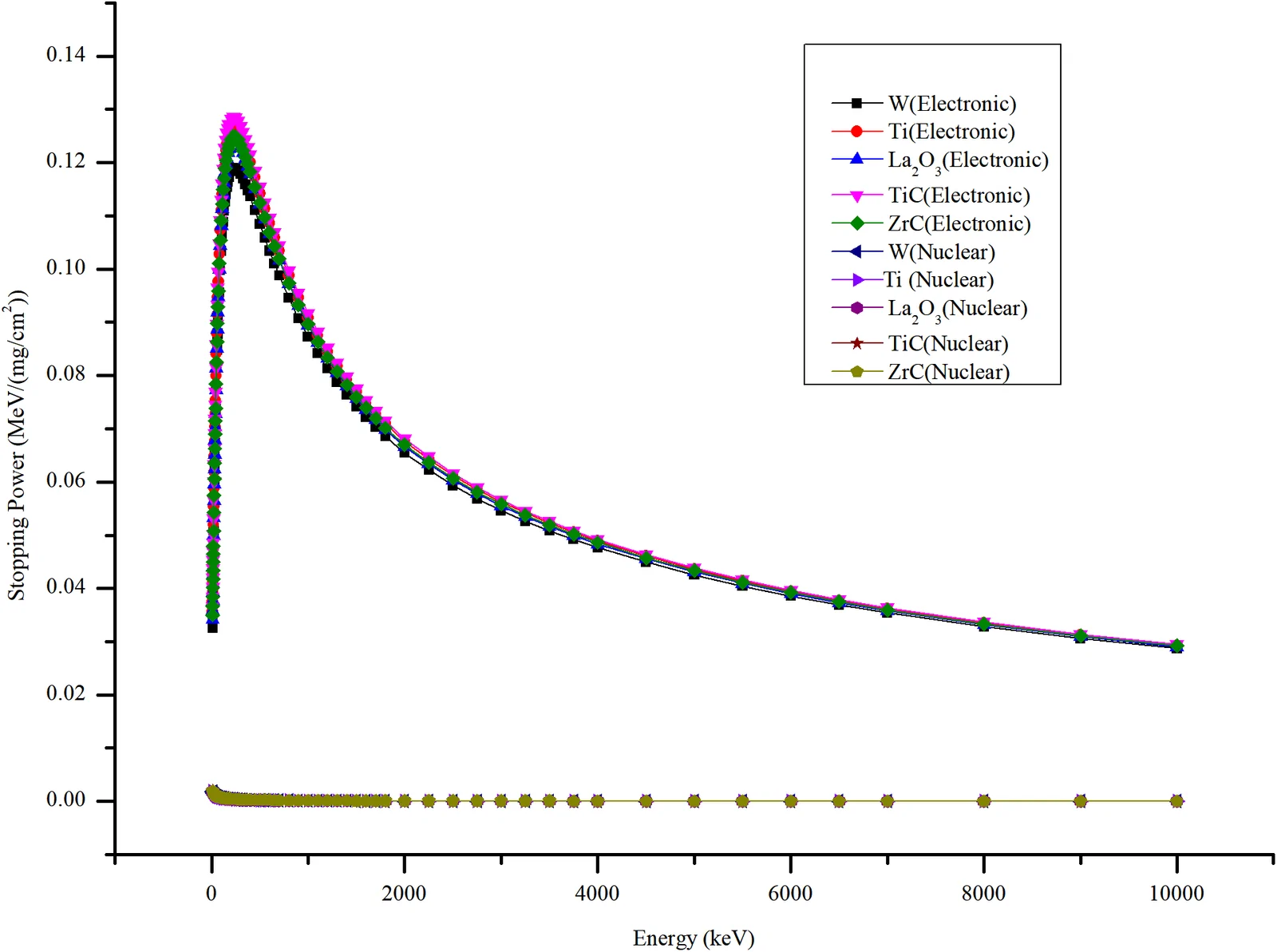
Stopping power as a function of incident energy.

### 3.2. Range

Fig 2 shows the range of deuterium ions in the five W-based targets as a function of incident energy. As shown in Fig 2, the range increases with increasing incident energy. When the energy is lower than 2000 keV, the ranges of deuterium ions in the five targets are nearly identical. When the energy exceeds 2000 keV, at the same incident energy the range in TiC homogeneously doped tungsten is slightly larger than that in the other targets, while the range in pure tungsten is the smallest. At an incident energy of 10 MeV, the maximum range is approximately 0.127 mm, indicating that deuterium ions mainly affect the near-surface region of tungsten-based targets.

**Fig 2:**
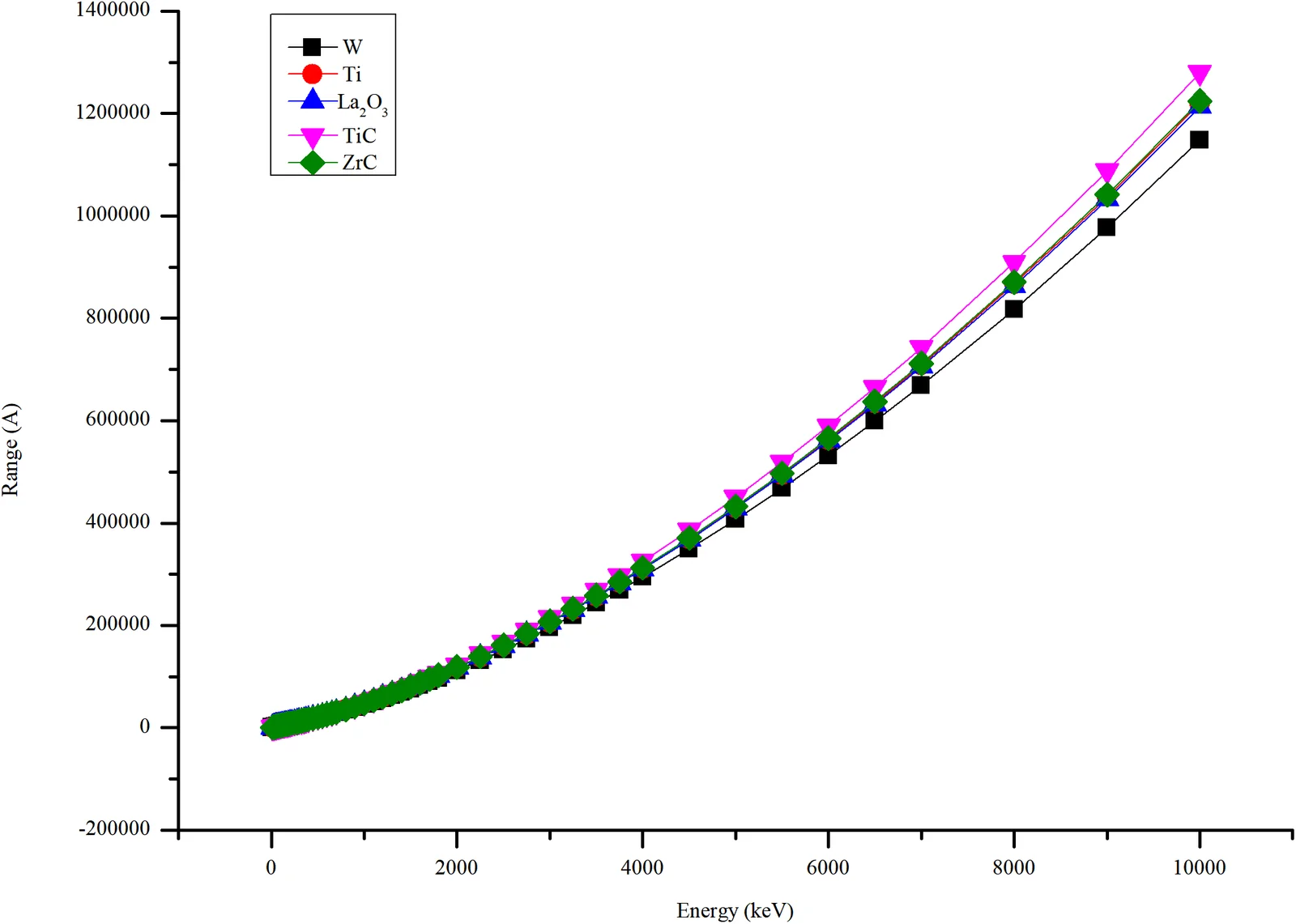
Range as a function of deuterium-ion incident energy.

### 3.3. Ballistic deposition distribution of deuterium ions in the target

As deuterium ions continuously collide with electrons and nuclei within the target, their kinetic energy dissipates gradually, and they eventually come to rest inside the target matrix. In this section, the static ion depth distributions output by SRIM are used to analyze the longitudinal distribution pattern of the stopping positions of deuterium ions at the initial stage of irradiation, and these results only reflect the instantaneous ballistic stopping behavior of deuterium ions upon implantation. It should be noted that the long-term deuterium retention capacity of tungsten-based materials, which dominates hydrogen embrittlement behavior, is determined by high-temperature thermodynamic processes. However, SRIM simulations do not incorporate thermally activated transport processes such as deuterium diffusion, defect trapping, and surface recombination under reactor operating temperatures. Therefore, the initial ballistic ion distributions obtained in this section cannot equivalently represent the actual long-term deuterium retention performance of the materials, yet these simulation results provide reference value for analyzing primary radiation damage.

Fig 3 presents the ballistic implantation depth distribution curves of incident deuterium ions for the five target materials irradiated by 100 keV deuterium ions. All five curves exhibit distinct concentration peaks, and the ballistic atomic number of stopped deuterium ions reaches its maximum at a depth of approximately 0.5 *μm*, which serves as the major deposition region for incident ions during primary irradiation. Overall, the ballistic implantation curves of deuterium ions for the five materials are highly consistent. Nevertheless, subtle differences can be observed in the region preceding the deposition peak: the ion concentration corresponding to the W-TiC composite is slightly lower than that of the other three doped tungsten specimens.

**Fig 3:**
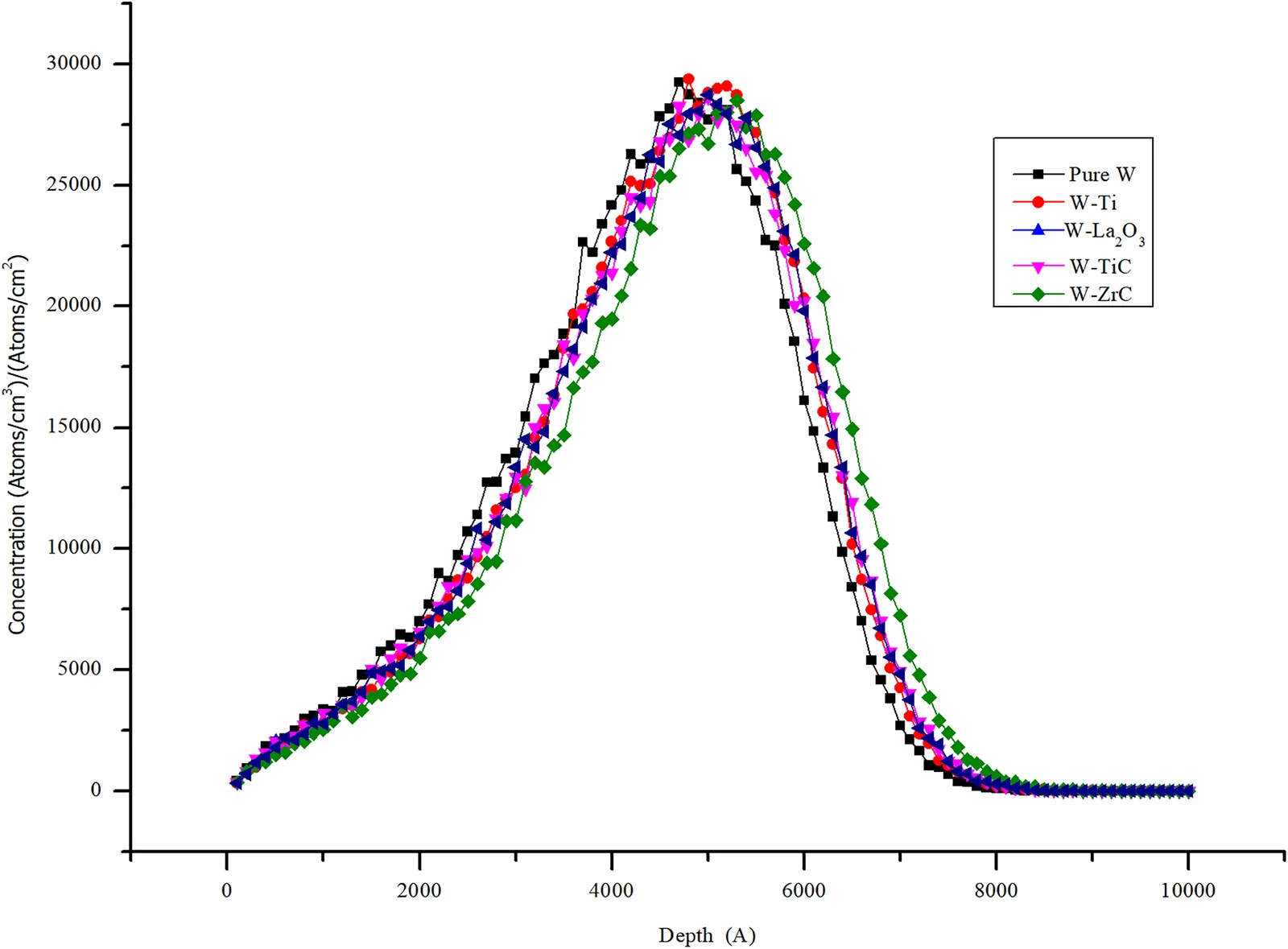
Ballistic implantation depth distribution profiles of deuterium ions in five targets.

### 3.4. Energy deposition of deuterium ions

Fig 4 shows the energy-deposition profile of 100 keV deuterium ions in the five targets. As shown in Fig 4, the energy deposition increases with depth to a peak and then drops sharply, which is consistent with the general energy-deposition behavior of energetic ions. Comparison among the five targets shows that pure W exhibits the highest energy-deposition peak, while all doped targets exhibit lower peaks, with the W–TiC target showing the lowest peak. This indicates that introducing dopants into tungsten can reduce the degree of energy-deposition localization, resulting in a more uniform energy distribution. A higher peak in pure W implies that the ion energy is more concentrated near the surface; larger near-surface energy deposition typically leads to more severe irradiation damage in the surface layer. Experimental measurements in Ref. [14] demonstrate that TiC- tungsten possesses far lower total deuterium retention and surface blister density than pure tungsten, and the trends obtained in this simulation are in complete qualitative agreement with the experimental observations in Ref. [14]. Therefore, doping tungsten is beneficial for reducing the localization of deuterium-ion energy deposition and mitigating irradiation damage, and the improvement in W–TiC is the most pronounced. This is consistent with the conclusion in Ref. [27] that dispersion strengthening can effectively improve the irradiation performance of tungsten-based targets.

**Fig 4:**
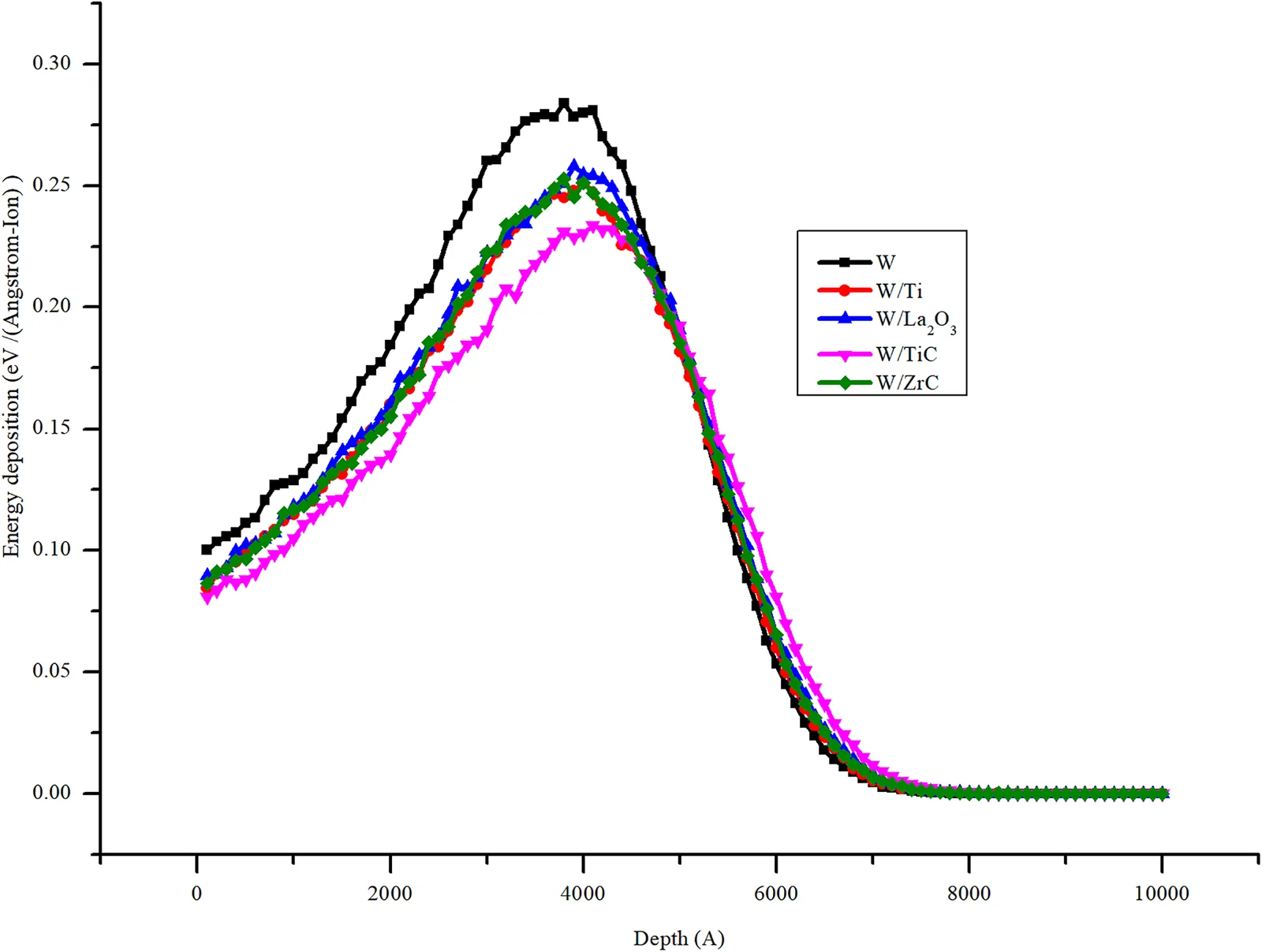
Energy-deposition profile of deuterium ions.

### 3.5. Displacement collisions of deuterium ions in the target

When energetic deuterium ions collide with target atoms, if the energy transferred to a target atom exceeds its displacement threshold energy, the atom will be displaced from its original lattice site to become an interstitial atom, leaving a vacancy at the original position. Such vacancy–interstitial pairs are known as Frenkel defects and are a typical form of irradiation damage. To quantify this type of damage, this study calculates the number of displacement collisions between a single deuterium ion and target atoms per angstrom of path length. A larger value indicates more frequent displacement events, more Frenkel defects, and more severe irradiation damage. Fig 5 shows the displacement-collision profile as a function of target depth for 100 keV deuterium ions in the five targets. As shown in Fig 5, the number of displacement collisions is the highest in pure tungsten and the lowest in W–TiC, while the other three doped targets lie in between. This confirms that introducing dopants into tungsten-based targets can effectively reduce displacement-collision frequency and thus mitigate irradiation damage, with W–TiC showing the most pronounced improvement, consistent with Ref. [27].

**Fig 5:**
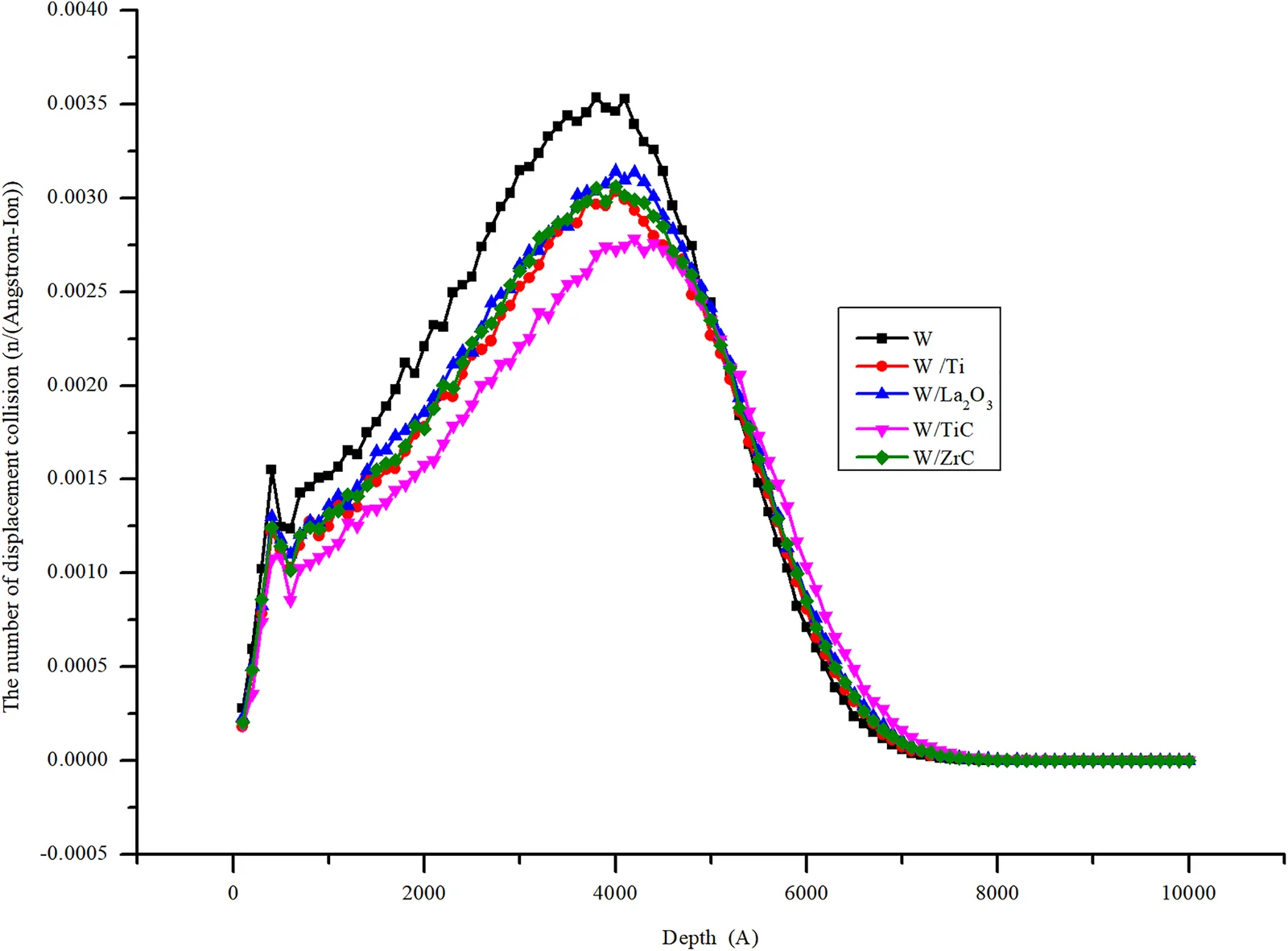
Displacement collisions as a function of target depth.

### 3.6. Vacancy production

Displacement collisions between deuterium ions and target atoms generate vacancy–interstitial pairs. Vacancies not only degrade thermal and mechanical properties, but also act as trapping sites for hydrogen and helium atoms, which may promote local accumulation of H/He. Fig 6 shows vacancy production as a function of target depth for 100 keV deuterium ions in the five targets. As shown in Fig 6, when the depth is less than 5000 Å, the vacancy number is the highest in pure W and the lowest in W–TiC, while the other three doped targets show similar vacancy levels. When the depth exceeds 5000 Å, the vacancy trends of the five targets are essentially consistent. Therefore, introducing dopant phases can effectively reduce vacancy production and improve the irradiation resistance of tungsten-based materials. Chen et al. [58] calculated the irradiation-induced vacancy distribution in W-La_2_O_3_ composites via SRIM simulations, and the variation trends of their curves are highly consistent with our results, which verifies the rationality of our calculations.

**Fig 6:**
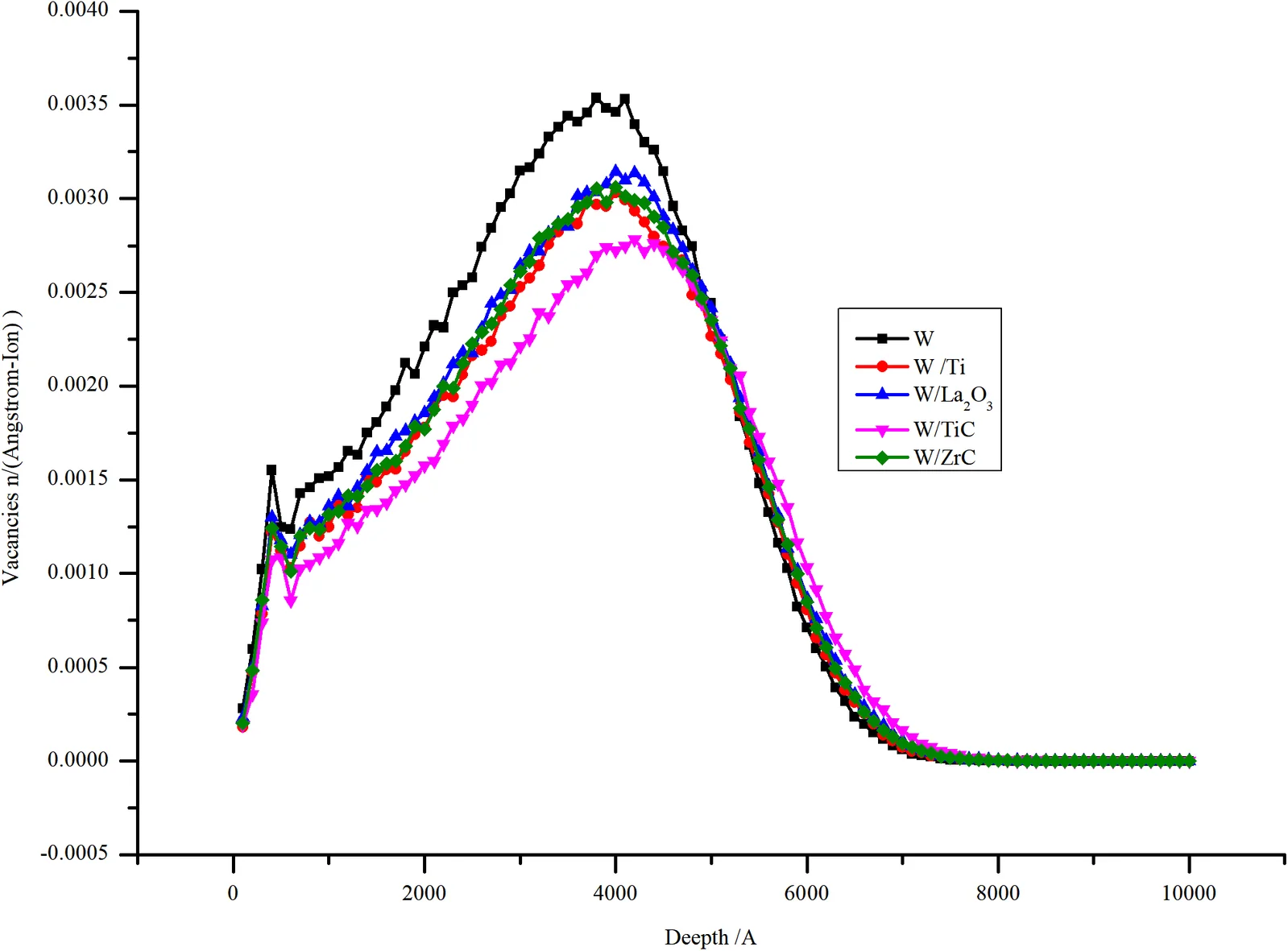
Vacancy production as a function of target depth.

It should be noted that SRIM does not account for the recombination and subsequent evolution of vacancies during collision cascades. Accordingly, the vacancy quantities calculated in this work cannot be directly used to quantitatively correlate long-term irradiation phenomena such as irradiation swelling, irradiation creep, hardening and bubble formation. These vacancy values only serve as a relative indicator of primary irradiation damage, rather than a prediction of surviving defect populations.

### 3.7. Effect of dopants content on irradiation damage

The above results were obtained for a dopant content of 3 wt.%. To further investigate the effect of dopant content, a composition gradient of 0.5 wt.%, 1 wt.%, 1.5 wt.%, 2 wt.%, 2.5 wt.%, 3 wt.%, 3.5 wt.%, 4 wt.%, 4.5 wt.%, 5 wt.%, 10 wt.%, and 15 wt.% was considered. The incident deuterium-ion energy was fixed at 200 keV, and the influence of dopant content on irradiation damage in tungsten-based targets was analyzed.

Unlike Sections 3.1–3.6, which use 100 keV deuterium ions to compare radiation performance of various dopants at a fixed doping content of 3 wt.%, this subsection selects 200 keV deuterium ions and establishes a wide gradient of dopant contents. Its primary research target is to quantitatively reveal the evolution regularities of vacancy, sputtering yield and ion range with rising doping fractions. The two energy conditions are utilized to realize two independent research perspectives: screening optimal dopant types and optimizing dopant doping contents, respectively.

#### 3.7.1. Vacancy production.

Vacancy production is one of the key parameters for evaluating irradiation damage. Fig 7 shows the vacancy number as a function of dopant content for four doped tungsten-based targets, meanwhile, the Poisson standard deviation of vacancies are also presented in the figure. As shown in Fig 7, vacancy production decreases with increasing dopant content for all four targets, but the extent of reduction differs among dopants. The order of effectiveness is Ti > La_2_O_3_ > TiC > ZrC. This indicates that all four dopants can reduce vacancy production, and vacancy number is approximately linearly negatively correlated with dopant content. Among them, Ti doping shows the most significant reduction in vacancy number, followed by La_2_O_3_. TiC shows a weaker effect, and ZrC shows the least effect. Meanwhile, as indicated by the height of the error bars, the Poisson standard deviation for the number of vacancies is nearly zero.

**Fig 7:**
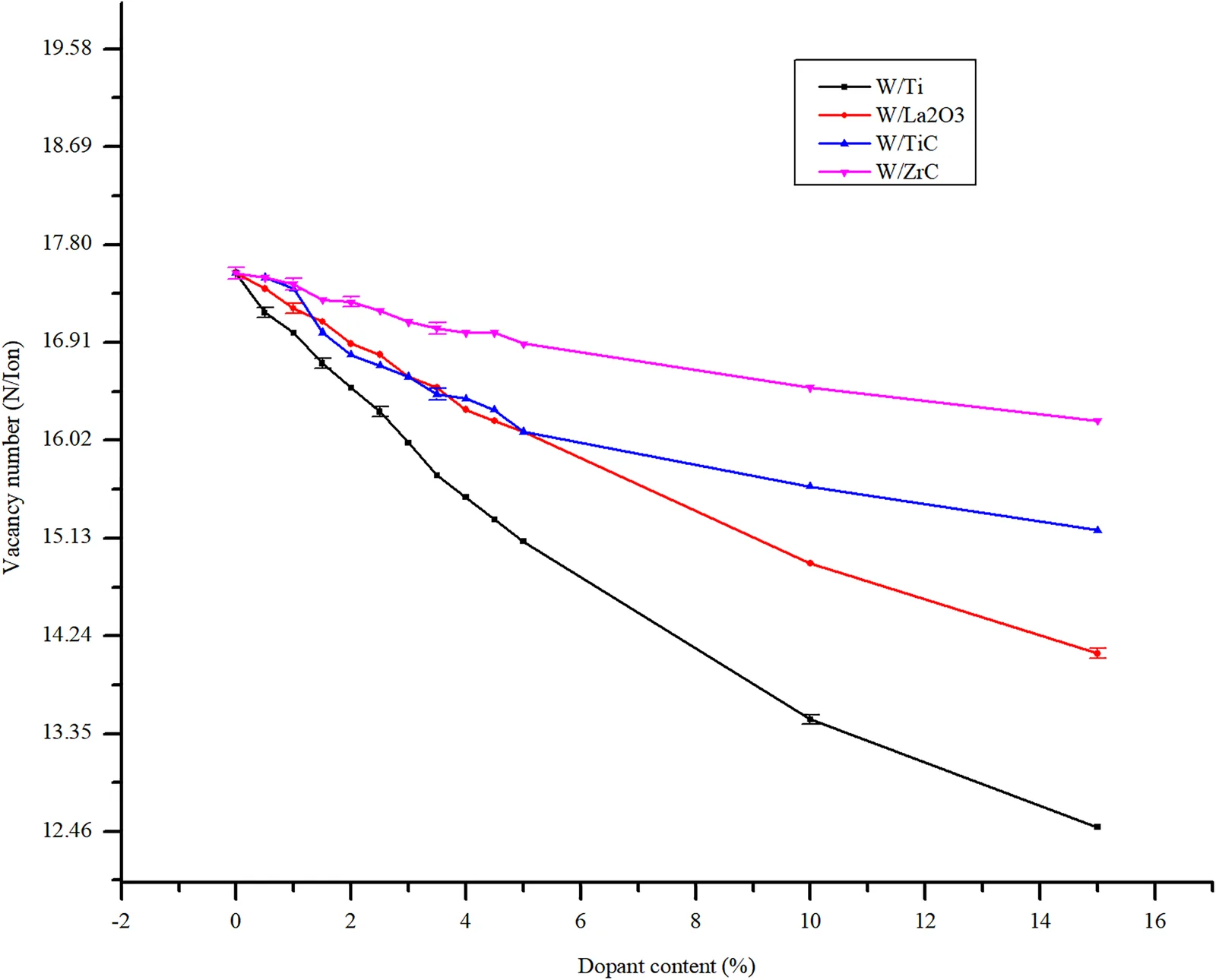
Vacancy production of four tungsten-based targets as a function of dopant content.

It should be specially noted that the conclusions obtained in this section and Section 3.6 cannot be directly compared. Section 3.6 counts the longitudinal distribution of local shallow vacancies (unit: vacancies/Å/ion), while this section calculates the overall average vacancies integrated over the full target thickness (unit: vacancies/ion). Moreover, the incident deuterium ion energies adopted are 100 keV and 200 keV respectively, and such differences in simulation conditions lead to distinct dopant performance rankings.

#### 3.7.2. Sputtering yield.

Sputtering causes first-wall atoms to enter the magnetically confined plasma region as impurities. Such impurities can deteriorate plasma confinement and reduce plasma quality, and in severe cases may lead to catastrophic consequences. Therefore, sputtering behavior is a key topic for plasma-facing materials. Against this backdrop, this work investigates the dependence of sputtering yield on dopant content for four types of tungsten-based targets, as shown in Fig 8. As illustrated in the figure, the sputtering yield fluctuates irregularly with increasing dopant fraction, and no distinct variation trend or regular pattern can be identified.

**Fig 8:**
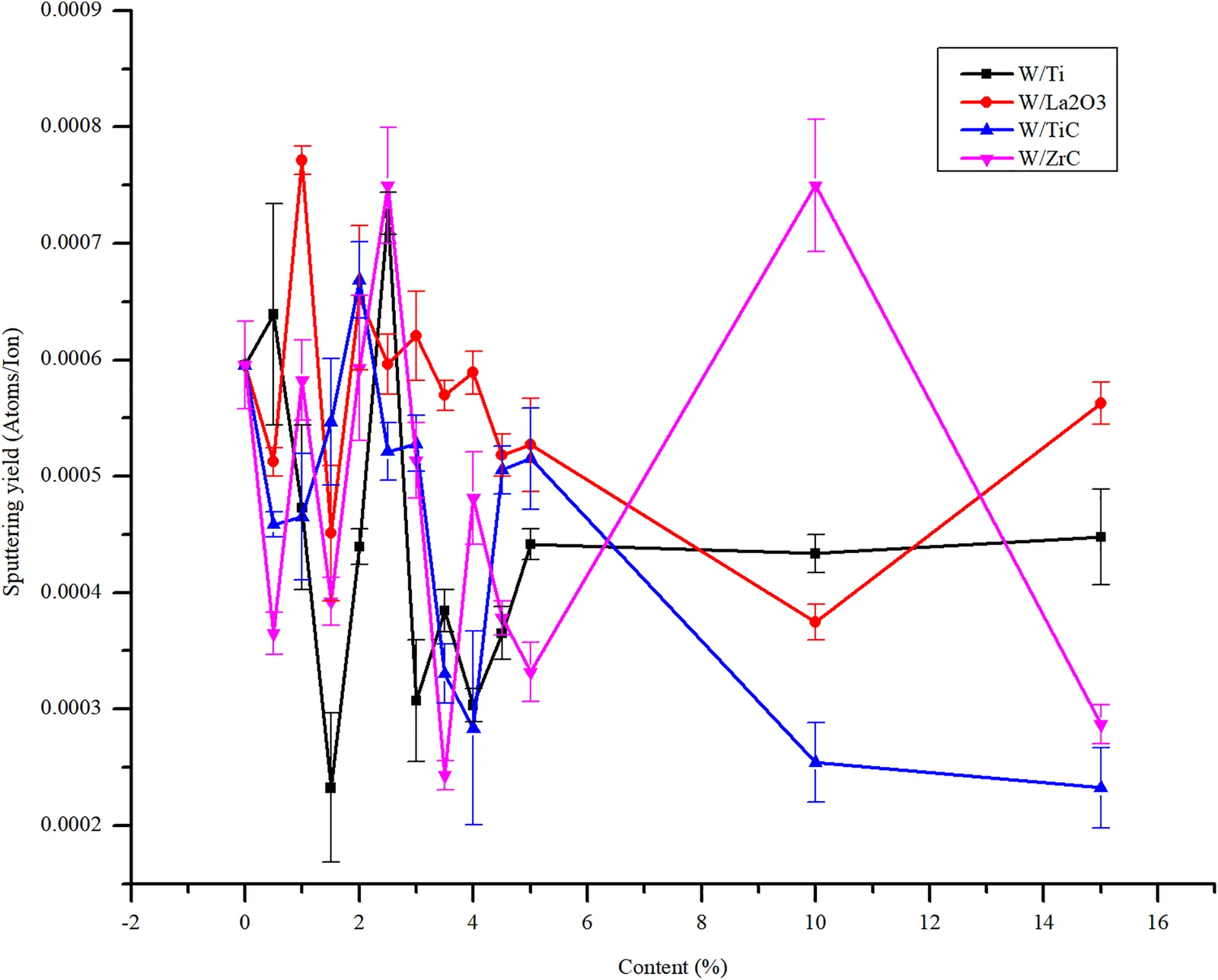
Sputtering yields and corresponding Poisson error bars of four doped tungsten composites as a function of dopant content.

To investigate the computational stability of SRIM outputs, comparative calculations with deuterium ion incident counts ranging from 80,000 to 120,000 were performed for all four dopant systems at each doping fraction. The results reveal that calculated parameters including energy deposition, displacement collision and vacancies exhibit excellent stability and barely change with the growth of incident ion numbers. In contrast, sputtering yields show dramatic fluctuations and poor computational repeatability. To reveal their variation trends, partial data are summarized in Table 2, which lists the sputtering yields, vacancies, their average values and corresponding Poisson standard deviations of four doped tungsten targets at dopant mass fractions of 1.5 wt.%, 4 wt.% and 10 wt.% under five incident ion counts between 80,000 and 120,000. As demonstrated in Table 2, merely varying the incident deuterium ion count while keeping other simulation parameters unchanged can lead to over twofold variation in sputtering yield, and the Poisson standard deviation can reach up to 30% of the mean sputtering yield under certain conditions. Such severe statistical fluctuation of SRIM-calculated sputtering yields makes it difficult to derive a credible evolutionary trend of sputtering yield. In contrast, the Poisson standard deviation for the number of vacancies is nearly zero.

**Table 2:** Sputtering yields, vacancies and Poisson errors for four doped W targets at different ion counts and dopant content.

Doping content (%)	Dopant		Number of incident ions					Average value	Error bar
			80000	90000	100000	110000	120000		
1.5	Ti	S	0.000275	0.000277	0.000390	0.000446	0.00045	0.000368	0.0000869
		V	16.7	16.7	16.7	16.7	16.7	16.7	0.0000000
	La_2_O_3_	S	0.000401	0.000411	0.000420	0.00049	0.000534	0.000451	0.0000580
		V	17.1	17.1	17.1	17.1	17.1	17.1	0.0000000
	TiC	S	0.000513	0.000500	0.000510	0.000618	0.000592	0.000547	0.0000543
		V	17.0	17.0	17.0	17.0	17.0	17.0	0.0000000
	ZrC	S	0.000425	0.000389	0.000390	0.000391	0.000367	0.000392	0.0000208
		V	17.3	17.3	17.3	17.3	17.3	17.3	0.0000000
4	Ti	S	0.000326	0.000378	0.000350	0.000527	0.000516	0.000419	0.0000951
		V	15.5	15.5	15.4	15.5	15.5	15.48	0.0447214
	La_2_O_3_	S	0.000576	0.000598	0.000600	0.000564	0.000608	0.000589	0.0000184
		V	16.3	16.3	16.3	16.3	16.3	16.3	0.0000000
	TiC	S	0.000163	0.000233	0.000320	0.000345	0.000358	0.000284	0.0000833
		V	16.4	16.4	16.4	16.4	16.4	16.4	0.0000000
	ZrC	S	0.000538	0.000500	0.000480	0.000446	0.000442	0.000481	0.0000399
		V	17.0	17.0	17.0	17.0	17.0	17.0	0.0000000
10	Ti	S	0.000613	0.000578	0.000590	0.000609	0.000591	0.000596	0.0000145
		V	13.5	13.5	13.5	13.5	13.5	13.5	0.0000000
	La_2_O_3_	S	0.000376	0.000366	0.000360	0.000372	0.000400	0.000375	0.0000153
		V	14.9	14.9	14.9	14.9	14.9	14.9	0.0000000
	TiC	S	0.000300	0.000277	0.000250	0.000227	0.000217	0.000254	0.0000345
		V	15.6	15.6	15.6	15.6	15.6	15.6	0.0000000
	ZrC	S	0.000726	0.000834	0.000780	0.000718	0.000691	0.000750	0.0000571
		V	16.5	16.5	16.5	16.5	16.5	16.5	16.5

S: Sputtering yield (Atoms/Ion)

V: Vacancies (Vacancies/Ion)

#### 3.7.3. Range.

Fig 9 shows the ion range as a function of dopant content for the four doped tungsten-based targets. As shown in Fig 9, the range increases approximately linearly with increasing dopant content, and the sensitivity to dopant content varies among dopant. The range in W–TiC is the most sensitive to dopant content; W–La_2_O_3_ and W–ZrC show similar trends; and W–Ti shows the mildest variation. At a fixed incident ion energy, a larger range indicates a smaller energy loss per unit distance in the target. Therefore, based on the range characteristics, deuterium ions lose the least energy per unit distance in W–TiC, corresponding to less severe ionization and displacement damage per unit path length, but with the deepest penetration depth. Experiments in Ref. [59] verify that Ti addition can reduce irradiation damage, yet its improvement effect is inferior to carbides. This matches perfectly the range order Ti < ZrC < TiC.

**Fig 9:**
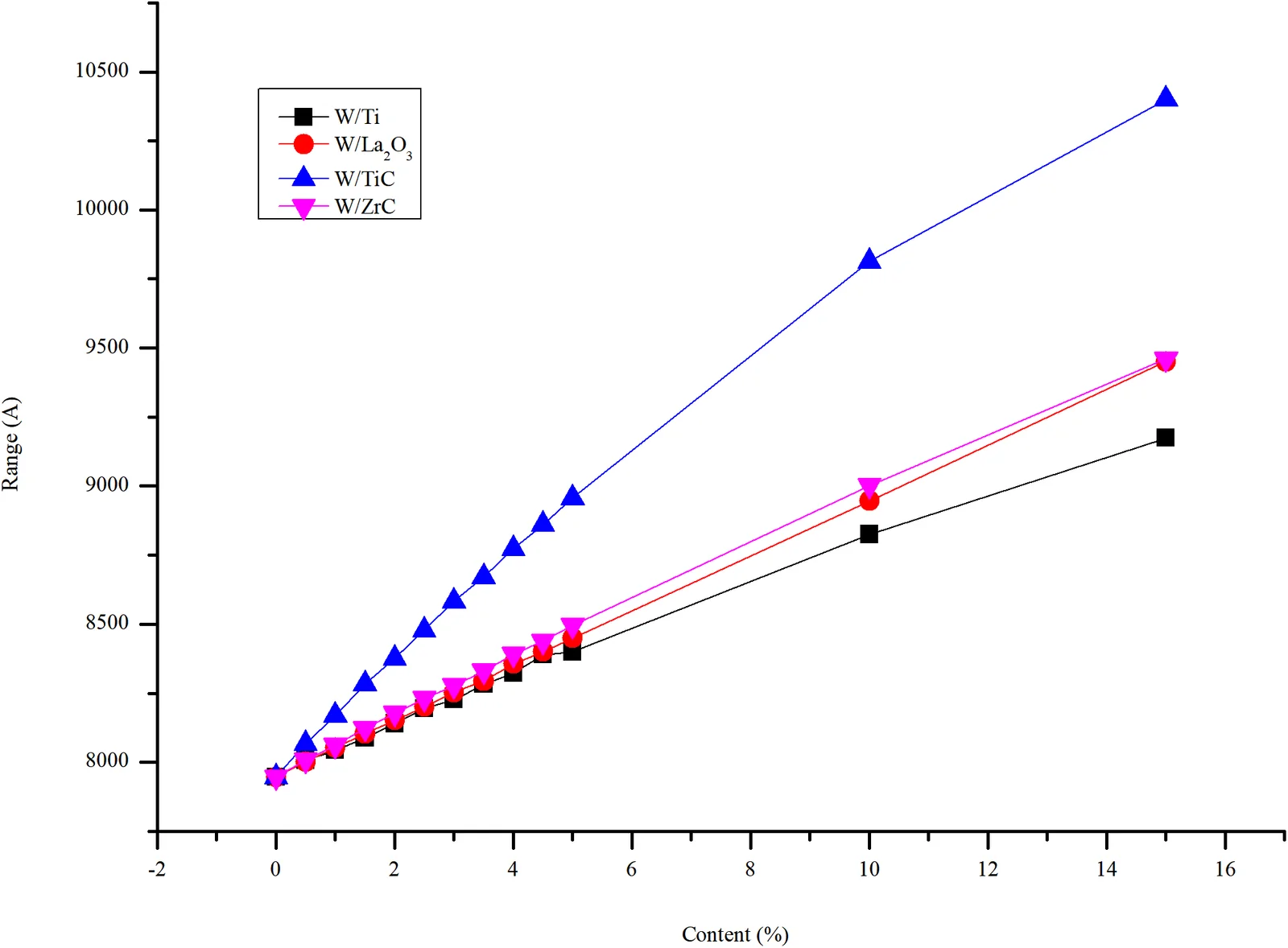
Range as a function of dopant content.

## 4. Conclusions

Deuterium irradiation severely degrades the performance of tungsten plasma-facing materials. SRIM simulations were carried out on pure W and four homogeneously doped W systems under two working conditions, with irradiation damage quantified from multiple dimensions. Main conclusions are as follows:

1. Simulations of pure tungsten and four homogeneously doped tungsten composites reveal that deuterium ions lose energy predominantly through electronic stopping with negligible nuclear stopping contribution across all incident energies. The ion range results demonstrate that irradiation damage induced by deuterium is concentrated within the near-surface region of targets.
2. At an incident energy of 100 keV and a fixed dopant fraction of 3 wt.%, the peak depth of deuterium deposition for all five materials is located around 0.5 μm. All doped systems exhibit lower energy deposition, displacement collision and vacancies than pure tungsten at the depths before their peak positions inside the targets,among which W–TiC shows the most pronounced effect
3. At an incident energy of 200 keV, increasing dopant content leads to an approximately linear reduction in vacancies, with Ti dopant showing the strongest vacancy inhibition effect, while the ion range rises linearly and W–TiC possesses the most significant range variation. Large sampling fluctuations of sputtering yield make it an unreliable indicator for evaluating doping performance. Overall, dopants effectively mitigate primary irradiation damage in tungsten.

Despite the reliable comparative trends derived from the above multi-dimensional quantitative analysis of irradiation damage, the SRIM simulation framework adopted in this work still carries several inherent methodological limitations that need to be explicitly clarified: ①All results of this study are obtained based on the homogeneous medium hypothesis of SRIM binary collision approximation, which carries inherent simulation limitations, the software simplifies multi-phase composite materials into average homogeneous media and cannot characterize local defect evolution induced by microstructural heterogeneities such as grain boundaries and particle-matrix interfaces; ②The full cascade calculation mode has numerical artifacts reported in literatures, only the relative comparison rules between materials are reliable, and there are systematic offsets for absolute damage values; ③Only two discrete incident energies of 100 keV and 200 keV are selected in this paper, and continuous energy gradient simulations are not carried out. The evolution law of irradiation damage with incident energy will be taken as the key research direction in the follow-up work; ④SRIM can only calculate primary irradiation damage on picosecond scale and cannot simulate defect clusters, thermal diffusion and long-term irradiation swelling evolution. Multi-scale modeling will be combined in the subsequent research to quantitatively analyze interfacial effects and long-time-scale damage.

In summary, the volume-averaged Monte Carlo quantitative results of primary irradiation damage in this paper systematically elaborate the regulation mechanisms of different dopants and different doping contents on deuterium irradiation behaviors of tungsten substrates. It is expected to provide quantitative simulation references for the compositional optimization of fusion plasma-facing tungsten materials and the design of anti-irradiation alloy formulations, and possess theoretical guiding value for the development of long-life first-wall components.

Finally, it should be emphasized that the results obtained at 100 keV (local vacancy depth distributions) and at 200 keV (depth-integrated vacancy numbers) reflect different irradiation conditions and damage metrics; the corresponding dopant rankings (W–TiC showing the most pronounced suppression of near-surface primary damage at 100 keV, and Ti showing the strongest vacancy inhibition at 200 keV) should therefore not be interpreted as a universal ranking of dopant effectiveness.

## Supporting information

S1 File Data of Nuclear stopping power, electronic stopping power and range. (XLSX)

S2 File Deuterium ion retention data. (XLSX)

S3 File Energy deposition data. (XLSX)

S4 File Displacement collision data. (XLSX)

S5 File Data for Vacancy distribution along the depth of the target. (XLSX)

S6 File Date for Vacancy number and error bars versus dopant content. (XLSX)

S7 File Data for Sputtering yield and error bars. (XLSX)

S8 File Data for range versus dopant contents. (XLSX)

S9 File Conversion of mass fractions to atomic fractions for SRIM input parameters. (DOCX)

S10 File Comparison of FDC and NRT damage-calculation modes for SRIM simulations of tungsten-based composites. (DOCX)
