# Supplementary material for: Study on radiation damage of tungsten-based targets induced by deuterium ions
Source: PLoS One. 2026 Oct 14;21(10):e0360154. doi: 10.1371/journal.pone.0360154 (PMC13667773; doi:10.1371/journal.pone.0360154)
Supplement: Supplementary file 1 [file pone.0360154.s009.docx]

**Input parameters at the calculation interface**

1. Calculation interface and parameter settings.

2. Calculation method for converting dopant mass fractions to integer atom‑count inputs for SRIM:

SRIM requires atomic fractions as input. We convert mass fractions to atomic fractions by assuming a total target mass of 100 g (97 g W matrix + 3 g dispersoid). Taking 3wt.% Ti-doped W as an example, the atomic counts of W (atomic mass = 183.8au) and Ti (atomic mass = 47.9au) are calculated by dividing mass by atomic weight, and the ratio of atom numbers is imported into SRIM. The detailed calculation formula:

$$\frac{97}{183.8}\text{：}\frac{3}{47.9}\approx5277\text{：}626$$

5277 (W) and 626 (Ti) are the atomic numbers entered into SRIM for 3 wt.% Ti-doped W. The same conversion scheme is adopted for all other dopant systems.

3. Mass fractions of dopants and corresponding converted atomic fractions used as SRIM input parameters for Ti/W, La_2_O_3_/W, TiC/W and ZrC/W composites.

| Mass fraction (wt.%) | Atomic fractions converted for SRIM input parameters | | | | | | | | |
| --- | --- | --- | --- | --- | --- | --- | --- | --- | --- |
|  | Ti/W | | La_2_O_3_/W | | | TiC/W | | ZrC/W | |
|  | W | Ti | W | La | O | W | Ti=C | W | Zr=C |
| 0.5 | 5413 | 104 | 5412 | 30 | 45 | 5412 | 82 | 5412 | 48 |
| 1 | 5386 | 209 | 5385 | 62 | 93 | 5385 | 163 | 5385 | 97 |
| 1.5 | 5359 | 313 | 5358 | 92 | 138 | 8358 | 244 | 5358 | 145 |
| 2 | 5332 | 418 | 5330 | 122 | 183 | 5330 | 325 | 5330 | 194 |
| 2.5 | 5305 | 522 | 5303 | 154 | 231 | 5303 | 406 | 5303 | 242 |
| 3 | 5277 | 626 | 5276 | 184 | 276 | 5276 | 488 | 5276 | 291 |
| 3.5 | 5250 | 731 | 5249 | 214 | 321 | 5249 | 569 | 5249 | 339 |
| 4 | 5223 | 835 | 5222 | 246 | 369 | 5222 | 650 | 5222 | 387 |
| 4.5 | 5196 | 939 | 5194 | 276 | 414 | 5194 | 732 | 5194 | 436 |
| 5 | 5169 | 1044 | 5167 | 306 | 459 | 5167 | 813 | 5167 | 484 |
| 10 | 4897 | 2088 | 4843 | 614 | 921 | 4843 | 1626 | 4843 | 969 |
| 15 | 4625 | 3132 | 4623 | 920 | 1380 | 4623 | 2439 | 4623 | 1453 |

4. Screenshot of output interface for Ti-doped W: Ti content = 3%, incident energy = 200 keV, incident ions = 80000. 
