# Supplementary material for: Study on radiation damage of tungsten-based targets induced by deuterium ions
Source: PLoS One. 2026 Oct 14;21(10):e0360154. doi: 10.1371/journal.pone.0360154 (PMC13667773; doi:10.1371/journal.pone.0360154)
Supplement: Supplementary file 2 [file pone.0360154.s010.docx]

**Comparison of FDC and NRT damage‑calculation modes for SRIM simulations of tungsten‑based composites**

1. **Vacancy-count comparison between FDC and NRT modes**

Table1. Vacancy-count comparison between FDC and NRT modes under different dopant contents

| Dopant Content(wt.%) | Number of vacancies (unit: vacancies/ion) | | | | | | | |
| --- | --- | --- | --- | --- | --- | --- | --- | --- |
|  | Ti/W | | La_2_O_3_/W | | TiC/W | | ZrC/W | |
|  | FDC | NRT | FDC | NRT | FDC | NRT | FDC | NRT |
| 0.5 | 17.2 | 9.7 | 17.4 | 9.8 | 17.3 | 9.9 | 17.5 | 9.9 |
| 1 | 17.0 | 9.7 | 17.2 | 9.8 | 17.2 | 10.0 | 17.5 | 9.9 |
| 1.5 | 16.7 | 9.7 | 17.1 | 9.7 | 17.0 | 10.1 | 17.3 | 9.9 |
| 2 | 16.5 | 9.7 | 16.9 | 9.7 | 16.8 | 10.1 | 17.2 | 10.0 |
| 2.5 | 16.2 | 9.6 | 16.8 | 9.7 | 16.7 | 10.1 | 17.2 | 10.0 |
| 3 | 16.0 | 9.6 | 16.6 | 9.7 | 16.6 | 10.2 | 17.1 | 10.0 |
| 3.5 | 15.7 | 9.6 | 16.5 | 9.6 | 16.5 | 10.2 | 17.1 | 10.0 |
| 4 | 15.5 | 9.5 | 16.3 | 9.6 | 16.4 | 10.3 | 17.0 | 10.0 |
| 4.5 | 15.3 | 9.6 | 16.2 | 9.6 | 16.3 | 10.4 | 17.0 | 10.1 |
| 5 | 15.1 | 9.7 | 16.1 | 9.5 | 16.2 | 10.4 | 16.9 | 10.3 |
| 10 | 13.5 | 9.4 | 14.9 | 9.3 | 15.7 | 10.8 | 16.5 | 10.4 |
| 15 | 12.4 | 9.2 | 14.1 | 9.1 | 15.5 | 10.9 | 16.2 | 10.4 |

Parallel simulations adopting both FDC and NRT modes were carried out for all tested dopant mass fractions, and the calculated vacancy data are summarized in Table 1. The absolute vacancy count predicted by the FDC mode is approximately 1.2–1.8 times higher than that obtained from the NRT mode. Notably, the NRT calculation exhibits very low sensitivity to dopant content variation: the total vacancy count hardly changes with the increase of dopant mass fraction, which cannot reflect the inherent correlation between vacancy magnitude and dopant content. In comparison, the FDC mode presents obvious compositional sensitivity; the vacancy quantity changes regularly with the dopant mass fraction, which supports the mechanistic analysis of vacancy evolution with dopant content in the main manuscript.

**2. Comparison of the two calculation modes and rationale for mode selection**

The selection of the FDC mode over the NRT mode for SRIM simulations in this work represents a deliberate decision made after comprehensive evaluation of the two calculation frameworks. The FDC and NRT modes differ in their underlying physical assumptions, the range of trackable collision events, and the types of output quantities they can provide. Since this study aims to investigate total vacancy production under ion irradiation as well as the compositional dependence of irradiation damage in tungsten-based composites, a calculation mode capable of capturing the full complexity of collision cascades and delivering physically meaningful defect statistics is required. The key considerations supporting this methodological choice are elaborated in the following sections:

2.1 Tracking of multi‑generation recoil atoms

The NRT‑based Quick Damage module only computes damage energy transferred from primary incident ions to target atoms and estimates total vacancies via analytical formulas. It does not track the transport and collision behaviours of secondary and tertiary recoil atoms. Multiple published irradiation‑simulation studies confirm that secondary‑ and tertiary‑recoil‑induced displaced vacancies occupy a substantial fraction of total defects. Ignoring these collision cascades will significantly underestimate vacancy yields and fail to reproduce cascade‑evolution characteristics faithfully.

2.2 Calculation requirement for sputtering yield

Sputtering yield is a key research parameter in this manuscript. The NRT Quick Damage mode cannot quantitatively output sputtering yield; accordingly, it cannot satisfy the analytical requirements of this study.

2.3 Sensitivity toward target‑material compositional variation

Parallel simulations with both modes were implemented for all tested dopant fractions. The comparative vacancy data are summarised in Table 1. The absolute vacancy number predicted by FDC is ~1.2–1.8 times larger than that from the NRT mode. Critically, the NRT mode exhibits extremely low sensitivity to dopant‑content variation: total vacancy counts barely fluctuate as dopant fraction changes, which prevents establishing the correlation law between vacancy magnitude and dopant content. In contrast, the FDC mode shows clear compositional sensitivity; vacancy quantities vary regularly with dopant proportion, which enables the mechanism analysis of vacancy evolution versus dopant content in the main text.

Furthermore, the NRT Quick Damage mode can only return the total vacancy number of the whole target, and cannot separate element‑resolved vacancy contributions originating from the tungsten matrix and different additive phases. Although element‑specific vacancy data are not presented in the main manuscript, FDC simulation outputs contain independent vacancy statistics for each atomic sublattice, which supports further defect‑origin analysis if needed.
